# Early Bioinformatic Implication of Triacidic Amino Acid Motifs in Autophagy-Dependent Unconventional Secretion of Mammalian Proteins

**DOI:** 10.3389/fcell.2022.863825

**Published:** 2022-05-13

**Authors:** Malay Ranjan Biswal, Sreedevi Padmanabhan, Ravi Manjithaya, Meher K. Prakash

**Affiliations:** ^1^ Computational Biology, Theoretical Sciences Unit, Jawaharlal Nehru Centre for Advanced Scientific Research (JNCASR), Bangalore, India; ^2^ Autophagy Laboratory, Molecular Biology and Genetics Unit, Jawaharlal Nehru Centre for Advanced Scientific Research (JNCASR), Bangalore, India

**Keywords:** unconventional protein secretion, autophagy, triacidic motif, LC3 interacting region, mammalian proteins

## Abstract

Several proteins are secreted outside the cell, and in many cases, they may be identified by a characteristic signal peptide. However, more and more studies point to the evidence for an “unconventional” secretion, where proteins without a hitherto unknown signal are secreted, possibly in conditions of starvation. In this work, we analyse a set of 202 RNA binding mammalian proteins, whose unconventional secretion has recently been established. Analysis of these proteins secreted by LC3 mediation, the largest unconventionally secreted dataset to our knowledge, identifies the role of KKX motif as well as triacidic amino acid motif in unconventional secretion, the latter being an extension of the recent implicated diacidic amino acid motif. Further data analysis evolves a hypothesis on the sequence or structural proximity of the triacidic or KKX motifs to the LC3 interacting region, and a phosphorylatable amino acid such as serine as a statistically significant feature among these unconventionally secreted proteins. This hypothesis, although needs to be validated in experiments that challenge the specific details of each of these aspects, appears to be one of the early steps in defining what may be a plausible signal for unconventional protein secretion.

## Introduction

Protein secretion is an essential cellular process. The first step in the translocation of secretory proteins across intracellular membranes and their final localization is the recognition of the “address tags” contained within the amino acid sequences of the proteins. In many cases of protein secretion, a specific configuration of 13–36 amino acids in the N-terminal region acts as a “signal peptide” and helps the translocation across the first membrane on the secretory pathway and thus universally controls the entry of all proteins to the secretory pathway in eukaryotes and prokaryotes. In eukaryotes, the signal peptide of a nascent precursor protein (pre-protein) directs the ribosome to the rough endoplasmic reticulum (ER) membrane and initiates the transport of the growing peptide chain across it (Devillers-Thiery et al., 1975; von Heijne, 1990). The pioneering work done in yeast and mammalian systems elucidated the mechanisms underlying eukaryotic classical secretory pathway (endoplasmic reticulum (ER)-Golgi-secretory vesicles) and demonstrated that proteins with signal peptides get secreted to the exterior which led to the 2013 Nobel Prize in physiology and medicine (Hata et al., 1993; Sollner et al., 1993; Barlowe et al., 1994; Bonifacino, 2014; Viotti, 2016). However, the conventional protein secretion (CPS) that employs the signal peptide alone is not responsible for the final destination of the mature protein; secretory proteins devoid of further address tags in their sequence are by default secreted to the external environment. Although signal peptides are not highly conserved, they have a common positively charged n-region, a hydrophobic h-region and a neutral, polar c-region (Nakai, 2000). The c-region contains a weakly conserved cleavage site recognized by membrane-bound signal peptidases. Before the translocation of the pre-protein across the ER membrane, a ribonucleoprotein called signal recognition particle (SRP) binds to the signal peptide emerging from the ribosome. Then the SRP-signal peptide-ribosome complex binds to the ER membrane *via* a SRP receptor (Blobel and Dobberstein, 1975).

Alternatively, unconventional protein secretion (UCPS) bypasses the conventional endoplasmic reticulum (ER)-Golgi route. Studies suggest four principal types of UCPS that can be further distinguished into non-vesicular and vesicular pathways (Rabouille et al., 2012; Rabouille, 2017). The non-vesicular pathways are further classified into Type I (e.g., FGF1) and Type II (e.g., yeast MATα). The vesicular pathways are mediated by Type III (e.g., Acb1) and Type IV (e.g., CFTR) mechanisms. Based on a recent classification, Type I is a pore-mediated translocation across the plasma membrane, Type II is an ABC transporter mediated secretion, Type III is an autophagosome/endosome-based secretion and Type IV is a Golgi bypass mechanism (Rabouille, 2017). The type III system has a unique feature as the autophagy process has the ability to form *de novo* vesicles, that have cargo specificity. One such selective form of autophagy that participates in UCPS is known as secretory autophagy (Jiang et al., 2013) wherein the cargo is secreted out instead of being degraded.

Unlike the classical secretory proteins that follow the canonical route of secretion (conventional protein secretion, CPS), the unconventionally secreted protein cargoes follow a plethora of divergent secretory mechanisms. There are no concrete studies on the motif analysis of UCPS. Even the signals that may trigger this UCPS are not clear. One of the early indications for what may be a possible signal in this fascinating unconventional secretion process, has only recently been discovered. The discovery of the diacidic motif, DE as the signal for UCPS of SOD1 (Cruz-Garcia et al., 2017) along with the context dependence of the presence of this motif in proximity with the charged, unstructured amino acids (Padmanabhan et al., 2018) might provide some clues. Similarly, motif-1 of the interleukin family is demonstrated to help in driving the unconventional secretion process (Zhang et al., 2020). On similar lines, the interaction between FGF2 and cell surface heparan sulfate is mediated by basic residues in the C-terminal part of FGF2 with K133 being an essential component of this binding motif (Temmerman et al., 2008; Nickel and Rabouille, 2009, 2008; Steringer et al., 2017).

With the DE motif as a potential UCPS export signal, the LIR containing proteins possess specific membrane associated receptors and the cells might use this in combination for the type III secretion. This can be resonated with the hypothesis that the UCPS cargo containing DE binds to a specific binding partner (Cruz-Garcia et al., 2018).

Predicting whether a protein undergoes a conventional secretion is a relatively well understood phenomenon. Several predictors, such as SecretomeP (Bendtsen et al., 2004) identify the signal peptide with very high accuracy. There are several other newer predictors such as the OutCyte (Zhao et al., 2019) and ExoPred (Ras-Carmona et al., 2021) which are meant to capture the unconventionally secreted proteins as well. These models based on artificial intelligence emphasize the accuracy rather than interpretability in terms of the potential signal-motifs. Further, the quality of the predictions itself may not be reliable as the models are trained on protein secretion data that is highly inhomogeneous. As such, a key to understanding the unconventional secretion signals, and mechanisms is the availability of the relevant high-quality data.

Increasing evidence implicates the role of autophagy proteins (ATGs) in the process of secretion. Indeed, genetic loss-of-function studies have revealed ATGs are required for the efficient secretion of inflammatory cytokines (Stow and Murray, 2013), extracellular release of bactericidal enzymes and tissue repair factors (Bel et al., 2017), extracellular vesicle production (Guo et al., 2017) and unconventional secretion of proteins lacking amino-terminal leader sequences (Rabouille et al., 2012). Some of the unconventional proteins that are shown to be secreted out include Acb1, IL1ß, TGFß (Schotman et al., 2008; Duran et al., 2010; Manjithaya and Subramani, 2010; 2011; Manjithaya et al., 2010; Dupont et al., 2011; Gee et al., 2011; Nilsson et al., 2013; Murrow et al., 2015; Son et al., 2015; 2016; Kortvely et al., 2016; Nuchel et al., 2018). As the process of secretory autophagy (Jiang et al., 2013) has been studied only in a small subset of cargoes the concept of microtubule associated protein Light Chain 3 (LC3) dependent EV loading and secretion (LDELS) from the secretomic studies has opened up more avenues to ponder upon the autophagy mediated secretory protein cargoes in detail (Leidal and Debnath, 2020; Leidal et al., 2020). The recent data on the 202 RNA binding proteins which are unconventionally secreted through an LC3-mediated pathway (Leidal et al., 2020) opens up the possibility of various analyses to understand UCPS. We performed bioinformatic analyses on this largest data set of autophagy mediated unconventionally secreted cargoes (202 RNA binding proteins) known till date to explore the possibility of identifying the signals that trigger unconventional secretion.

## Methods

### Sequence Curation

The 202 unconventionally secreted proteins used in the analysis are obtained from the set of proteins proved to be secreted by LC3-mediated mechanism in the analysis of Leidal et al. (Leidal et al., 2020). The set of 1576 conventionally secreted proteins are obtained from the reference set used for training in SecretomeP (Bendtsen et al., 2004) database (http://119.3.41.228:8080/SPRomeDB/download_enabled.php). For convenience, these data sets are also provided in our Supplementary Data (https://github.com/malayrb/Thesis/tree/main/Ch7).

### Discriminatory Motif Analysis

Discriminatory motif (DiMotif) analysis (Asgari et al., 2019) of a set relative to the Swiss-Prot reference was performed using the code: https://github.com/ehsanasgari/dimotif/blob/master/notebook/DiMotif_step_by_step_example.ipynb.

### Motif Search Analysis

In addition to the DiMotif analysis, we performed a motif search using our script to analyse the differential occurrence of the motifs. The analyses presented in this work are based on 3 amino acid motifs, and which can result in 4200 combinations or 8000 combinations respectively with and without considering the mirror symmetry of the motifs. The proteins from the conventionally secreted and the LC3-mediated groups were scanned for these motif combinations, and the presence or absence of the motif was noted. Similarly, scripts were also used for analysing 4 amino acid motifs as well as the LIR motif (WXXL) (Noda et al., 2010; Jacomin et al., 2016).

## Results

### Acidic Motifs Top the Differential Motif Analysis

The presence of a signature signal sequence is common in protein sorting. To identify the presence of a signal sequence in the 202 RNA binding proteins, we performed two different analyses:

#### Discriminatory Motif Analysis

To capture the unique signature in the 202 LC3 interacting proteins (UCPS-ATG dataset), they were compared against 20,117 proteins from the Swiss-Prot database using the DiMotif server (Asgari et al., 2019). This discriminatory motif analysis is meant to identify the motifs which were significantly represented in a chosen set, relative to all the proteins from the Swiss-Prot database. The most significant motifs identified by this analysis are (details shown in Supplementary Table S1A): EEE, DD, DED, DE, AK, KKE, KK, KT, AKK, KE. These discriminatory motifs have two as well as three amino acids.

#### Custom Motif Analysis

In addition to the above-mentioned analysis from DiMotif server, we also performed a custom motif search comparing the LC3 interacting proteins with the database of conventionally secreted proteins used for training the SecretomeP (Bendtsen et al., 2004). In this analysis, all possible motifs of 2, 3 and 4 amino acids were combinatorially generated and a systematic search for them was performed in the LC3 interacting UCPS-ATG dataset (positive-set), and the conventionally secreted proteins (negative-set) (Bendtsen et al., 2004). The implicit assumption being that the conventionally secreted proteins are not secreted through the LC3-mediated pathway. Comparing the motifs in the positive and the negative sets, the top ten differentiating proteins were identified after imposing a constraint that the motif must occur at least 30% more often among the proteins in the positive-set than in the negative-set. The occurrence of a motif in the protein, rather than the number of its occurrences in the same protein, was considered important. In this differential analysis, three amino acid motifs had the highest difference between the two sets, while two or four amino acid motifs did not appear to differentiate the two sets significantly to appear among the top differentiators. The three amino acid motifs with the highest difference between the two sets are: EEE, KKS, AEK, AKK, KKR, KEL, DEE, KAL, EKL, KER (details in Supplementary Tables S1B,C). As may be seen, most of the differentiating motifs are charged, with the triacidic motif at the top.

### Acidic Motifs Appear in the Proximity of LIR Motifs

The transport of the specific set of proteins analysed in this work is mediated through the LC3 domains. We identified all the LC3 Interacting Regions (LIR) in each protein by performing a search for WXXL motif (Noda et al., 2010; Jacomin et al., 2016), and studied the frequency of occurrence of the different 3 amino acid motifs in the proximity of LIR. As the structural information of these proteins is sparse, we restricted the primary analysis to sequence-based proximity and wherever the structural information was available, the structural proximity check was subsequently checked for. The most commonly occurring sequences in the proximity of the LIR regions are: KEL, EEL, ALE, KAL, DEE, EKL, AEE, EEE, EEK (details in Supplementary Table S2).

### Phosphorylatable Amino Acids Occur Preferentially in the UCPS Proteins

Since the unconventional secretion is usually activated under conditions of stress, we explored the possibility that a post-translational modification may be required for its activation. We searched for the presence of serine, threonine, or tyrosine within 3 amino acid positions from the differentiating motifs. For almost all the reference motifs we analysed, the S/T/Y amino acid in the proximity of the motifs occurred preferentially among the proteins from the positive set (Supplementary Table S3).

## Discussion

### Triacidic Motif is Potentially a Signal for UCPS in Mammalian Cells

The discriminatory motifs identified relative to the Swiss-Prot database and the conventional protein secretion dataset were re-grouped to identify the common patterns among them. Two major patterns emerge among these three amino acid motifs: triacidic motifs (EEE/DDD/DEE/etc) occurring in 160 of the 202 from the positive set, and 625 of the 1576 in the negative set, and basic motifs (KKX) occurring in 187 of the 202 from the positive set and 796 of the 1576 in the negative set. Considering either of these triacidic or KKX motifs as a signal, the difference in the proportion between the positive and the negative data sets is statistically significant (*p* < 0.0002 in a Z-test).

**FIGURE 1:**
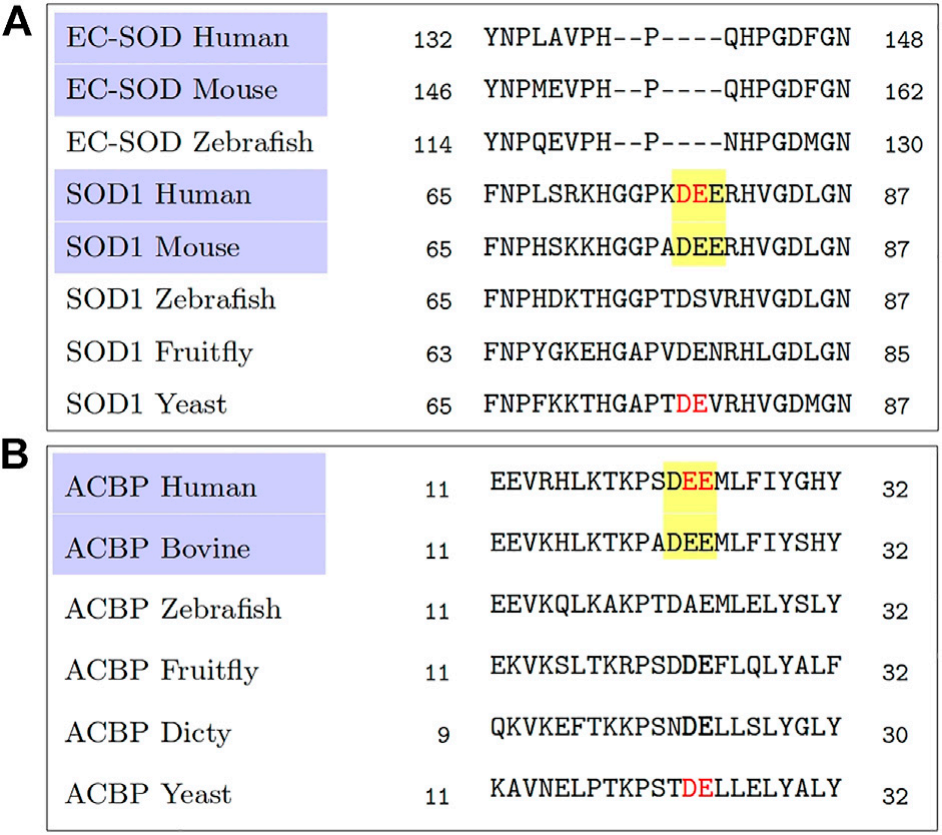
A reanalysis of the multiple sequence alignments obtained from Cruz-Garcia et al. (2017). A comparison among the mammalian sequences, highlighted in purple, shows a common triacidic motif DEE (highlighted in yellow), rather than a diacidic motif (shown in red colored text) when comparing sequences across all species. **(A)** Multiple sequence alignment from homologs of SOD1. **(B)** Multiple sequence alignment from homologs of ACB1.

Of these two statistically significant observations, the triacidic motif, by coincidence, happens to be an extension of the observation of the diacidic motif (Cruz-Garcia et al., 2017) and our earlier attempt to find the context in which the diacidic motif appears (Padmanabhan et al., 2018). In fact, a quick reanalysis of the multiple sequence alignments from the homologs of SOD1, Acb1 (Cruz-Garcia et al., 2017) by focusing on the mammalian sequences alone shows that they all have a common triacidic motif (Figure 1). However, despite the statistical evidence over the 202 proteins for the possibility of KKX as a UCPS signal, it is present neither in SOD1, nor in Acb1. Since there is very limited data on unconventionally secreted proteins, we consider the independent finding of the triacidic motif in an already experimentally validated data set as evidence in support of our finding. Needless to say, the role of which KKX as well as the other features possibly contributing to the signal, as described below require further computational as well as experimental investigations. Further, in the positive set which is derived from LC3-mediated secretion (Leidal et al., 2020), the validation of the triacidic or KKX motifs for other types of unconventional secretion will also require investigation.

### Phosphorylation May Be Activating the Signal

Unlike conventional secretion, the UCPS is activated under conditions of stress, suggesting the possibility that post-translational modifications may play a role in activating the signal. In continuation of the hypothesis that the triacidic or KKX motifs may be the “signal”, we explored the possibility that the amino acids S/T/Y in the proximity are responsible for activating this signal. S/T/Y amino acids in the proximity of triacidic motifs appeared in 133 of the 202 proteins from the positive-set, and in 422 of the 1576 proteins from the negative set. Similarly, S/T/Y amino acids in the proximity of KKX appeared in 170 of the 202 proteins from the positive set, and in 603 of the 1576 proteins from the negative set. The statistical significance of the difference between the two sets remains high (*p* < 0.00001 in a Z-test).

### LIR Motif in the Proximity of Triacidic Motif is Discriminatory

**FIGURE 2:**
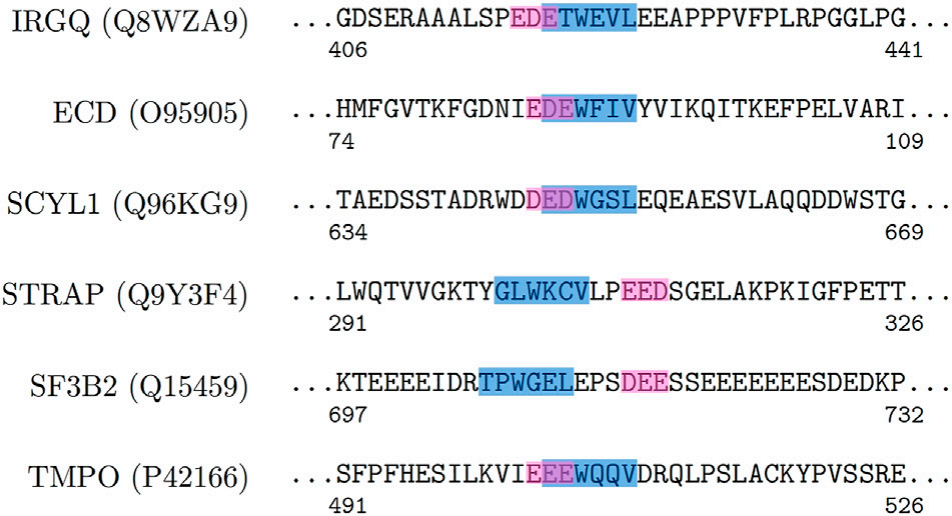
An analysis of the sequence-proximity of the triacidic motif with the LIR motif among some of the proteins from the class I of the UCPS-ATG data set is shown.

**FIGURE 3:**
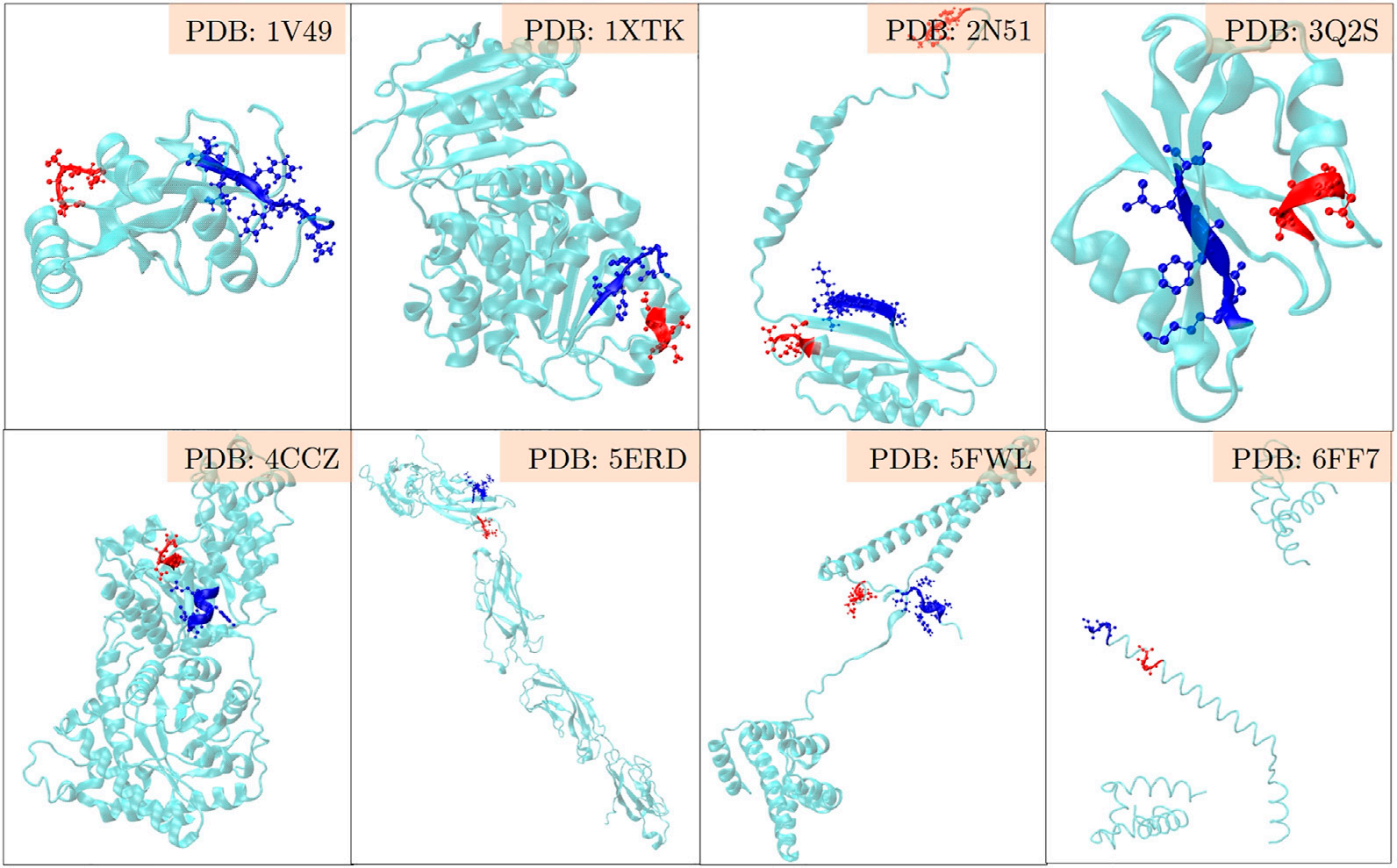
An analysis of the structural-proximity of the triacidic motif with the LIR motif among the proteins from the class I of the UCPS-ATG data set for which structures are known is shown. The blue and red colors indicate the LIR and triacidic motifs. For convenience, only the closest pair is shown and other occurrences of LIR or triacidic motifs are not shown.

**FIGURE 4:**
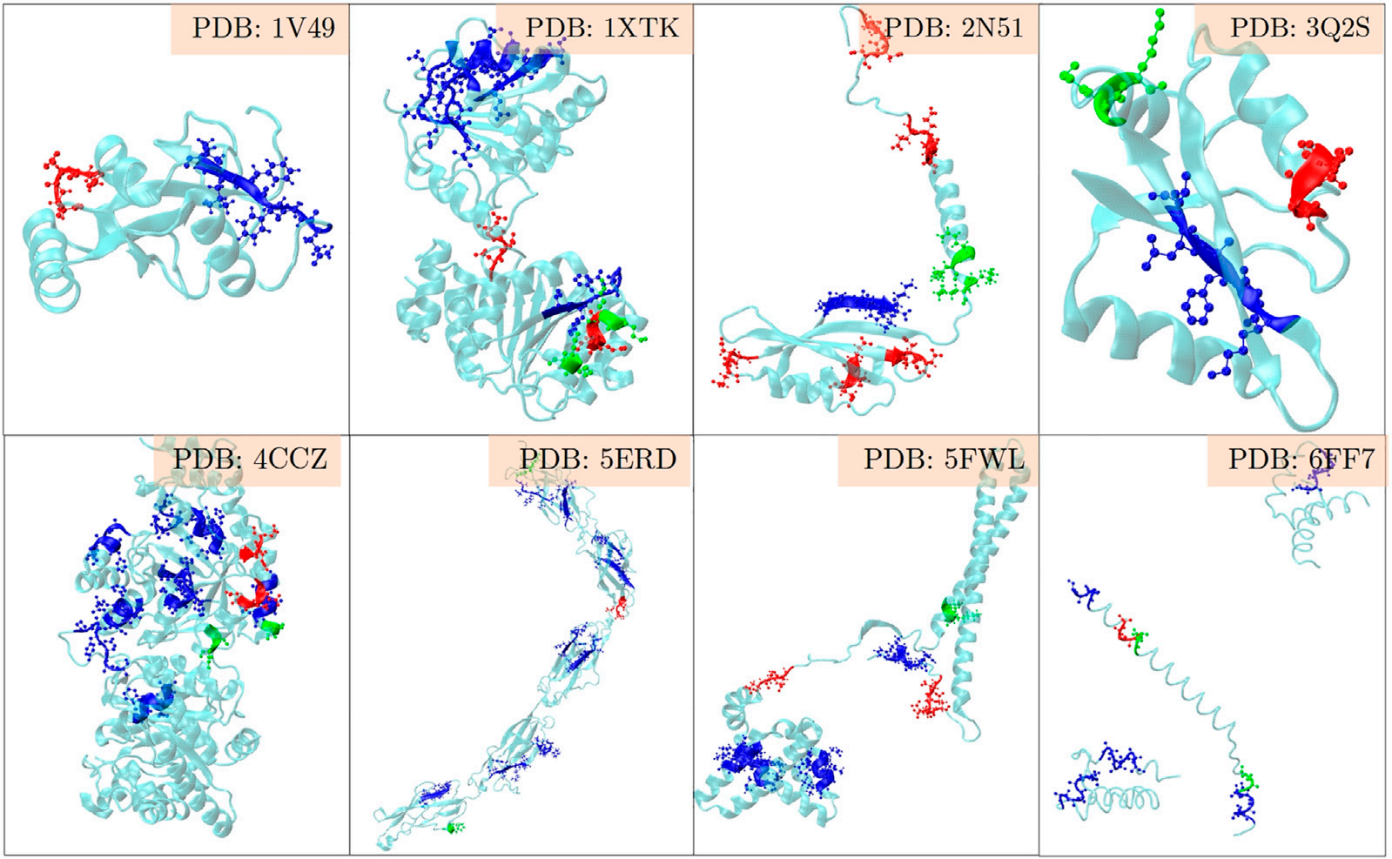
An analysis of the structural-proximity of the triacidic as well as the KKX motifs with all LIR motifs occurring among the proteins from the class I of the UCPS-ATG data set for which structures are known is shown. The blue, green and red colors indicate the LIR, KKX and triacidic motifs. One may notice that in some structures LIR is close to the triacidic motif, and in others to the KKX motif.

The positive set being analysed here, is about the set of proteins where LC3 conjugation machinery is involved in their secretion (Leidal et al., 2020). However, the LIR motifs are present in abundance in both the positive and the negative sets, making them non-discriminatory. To investigate beyond the statistical averages from the 202 proteins, and to obtain fine-grained insights into the role of LIR and the triacidic motifs, we analysed the 31 class I proteins from the positive-set which were secreted in all three replicates in a statistically significant way. Among them, 6 proteins had LIR motif within 3 amino acids of the triacidic motif (Figure 2) along the sequence. From the remaining proteins, structural information was available only for 8 of them and in all of them the LIR region was within a structural proximity (Figure 3), if not a sequential proximity of 10 Å from triacidic motifs (Supplementary Table S4). Coincidentally, in the cases where the structural proximity between the LIR and the triacidic motifs was not seen, it could be seen with the KKX motifs (Figure 4), underscoring the possible complementarity between the triacidic and KKX motifs in signaling the UCPS.

### Deriving the Hypothesis for the Signal for UCPS

Given the importance of unconventional protein secretion, it is pivotal to identify the signals that trigger it, if such signals exist. The key to building hypotheses is to work with highly reliable data, preferably from fewer sources to avoid any biases in the experimental protocols. In this work, we chose to work with a very specific data set from the LC3 machinery driven protein secretion with 202 proteins, and to build a few hypotheses on what may be the signal for the unconventional secretion. The presence of three amino acid motifs, triacidic or KKX, appears recurrently in the set of 202 UCPS proteins, significantly more than it occurred either in the conventionally secreted proteins or in the Swiss-Prot database. Although the 202 proteins are believed to be secreted by the LC3 dependent pathway, 5 of these proteins do not have an LIR motif that can interact with the LC3 region. Interestingly even in these proteins, triacidic motif in the proximity of a phosphorylatable amino acid is a common occurrence. Among the proteins that had the LIR motif, it was found mostly in the sequence or a structural proximity from the triacidic or the KKX motifs. Thus, it appears that triacidic or KKX amino acid motifs in the proximity of LIR and/or phosphorylatable amino acids may play a significant role in triggering the unconventional secretion. This result was also validated in the independently curated dataset of unconventionally secreted proteins from other mammalian cells (Padmanabhan et al., 2018), where among the 26 mammalian proteins that are secreted unconventionally, 5 of them had triacidic motifs within a 5 amino acid proximity of LIR. 9 of the remaining proteins where there was no sequence proximity, but the structures were available, had LIR motifs within 10 Å of the triacidic motif, and three other structures had them within 15 Å. It will be very interesting to see if this hypothesis can be validated and refined with new experiments in which mutant constructs are designed to challenge each of these aspects of the composite hypothesis–triacidic, KKX, proximity of LIR, proximity of serine amino acid - are developed.

## Conclusion

In conclusion, we explored the plausible signals for a very fundamental cellular process - unconventional protein secretion. The field is still in its nascent stages compared to conventional protein secretion where the signals as well as the mechanisms are clearly identified. Exploiting the recent experimental findings of a large set of unconventionally secreted proteins, we could perform bioinformatic analyses as well build hypotheses on the potential role of triacidic amino acids or KKX motif in the proximity of LIR region and phosphorylatable amino acids. As the next steps, we will be exploring collaboration with the relevant experimental groups to validate these hypotheses as well as explore the possibility of deciphering the patterns using interpretable deep-learning methods on the same datasets.

## Data Availability

The original contributions presented in the study are included in the article/Supplementary Material, further inquiries can be directed to the corresponding authors.
