# Supplementary material for: Early Bioinformatic Implication of Triacidic Amino Acid Motifs in Autophagy-Dependent Unconventional Secretion of Mammalian Proteins
Source: Front Cell Dev Biol. 2022 May 13;10:863825. doi: 10.3389/fcell.2022.863825 (PMC9136135; doi:10.3389/fcell.2022.863825)
Supplement: Supplementary file 1 [file Table1.docx]

**Supplementary Table S1A.** Discriminatory motif analysis of the UCPS-ATG set relative to the SwissProt database. The E-value denotes the significance level of the hypothesis that the sequence appears selectively in the UCPS-ATG dataset, and with these extremely small values of it, it is practically the same as the p-value.

| **Top motifs** | **E-value** |
| --- | --- |
| EEE | 4.47E-34 |
| DD | 1.40E-29 |
| DED | 9.08E-27 |
| DE | 2.14E-26 |
| AK | 6.32E-26 |
| KKE | 4.56E-24 |
| KK | 3.65E-23 |
| KT | 4.23E-23 |
| AKK | 8.80E-23 |
| KE | 9.78E-23 |
| DDD | 2.98E-22 |
| EK | 8.83E-22 |
| RGRG | 1.25E-21 |

**Supplementary Table S1B.** A summary of the motifs that occur at least 30% more in the UCPS-ATG dataset than in the CPS dataset. The total number of proteins in both the sets as well as the percentage of occurrence in them are both shown.

| **Motifs** | **%UCPS-ATG (202)** | **%CPS (1576)** |
| --- | --- | --- |
| EEE | 50.99 | 13.39 |
| KKS | 45.54 | 11.36 |
| AEK | 47.52 | 13.96 |
| AKK | 44.55 | 11.10 |
| KKR | 41.58 | 8.63 |
| KEL | 54.95 | 22.08 |
| DEE | 51.98 | 19.23 |
| KAL | 51.98 | 19.35 |
| EKL | 51.98 | 19.54 |
| KER | 42.08 | 9.77 |
| EKK | 43.56 | 11.80 |
| EEK | 49.50 | 17.83 |
| EKR | 43.07 | 12.18 |
| AEE | 51.98 | 21.13 |

**Supplementary Table S1C.** Comparison of occurrence of the triacidic motifs and KKX motifs in the unconventionally secreted proteins as well as in the conventionally secreted proteins.

| **Motifs** | **UCPS-ATG (202)** | | **CPS (1576)** | |
| --- | --- | --- | --- | --- |
|  | Number | % | Number | % |
| DDD | 49 | 24.26 | 103 | 6.54 |
| DDE | 88 | 43.56 | 236 | 14.97 |
| DED | 69 | 34.16 | 143 | 9.07 |
| DEE | 105 | 51.98 | 303 | 19.22 |
| EDE | 75 | 37.13 | 174 | 11.04 |
| EEE | 103 | 50.99 | 211 | 13.39 |
| KKX | 187 | 92.57 | 796 | 50.51 |
| Any Triacidic Motif | 160 | 79.21 | 625 | 39.66 |
