# Supplementary material for: Early Bioinformatic Implication of Triacidic Amino Acid Motifs in Autophagy-Dependent Unconventional Secretion of Mammalian Proteins
Source: Front Cell Dev Biol. 2022 May 13;10:863825. doi: 10.3389/fcell.2022.863825 (PMC9136135; doi:10.3389/fcell.2022.863825)
Supplement: Supplementary file 2 [file Table2.docx]

**Supplementary Table S2** Propensity of occurrence of the various motifs in the proximity of LIR motif in the unconventionally secreted proteins as well as in the conventionally secreted proteins.

| **Motifs** | **UCPS-ATG (202)** | | **CPS (1576)** | |
| --- | --- | --- | --- | --- |
|  | Number | % | Number | % |
| KEL | 20 | 9.90 | 92 | 5.84 |
| EEL | 17 | 8.42 | 118 | 7.49 |
| ALE | 16 | 7.92 | 54 | 3.43 |
| KAL | 15 | 7.43 | 57 | 3.62 |
| DEE | 5 | 2.48 | 30 | 1.90 |
| EKL | 19 | 9.41 | 91 | 5.77 |
| AEE | 17 | 8.42 | 32 | 2.03 |
| EEE | 8 | 3.96 | 20 | 1.27 |
| EEK | 12 | 5.94 | 25 | 1.59 |
