# Supplementary material for: Early Bioinformatic Implication of Triacidic Amino Acid Motifs in Autophagy-Dependent Unconventional Secretion of Mammalian Proteins
Source: Front Cell Dev Biol. 2022 May 13;10:863825. doi: 10.3389/fcell.2022.863825 (PMC9136135; doi:10.3389/fcell.2022.863825)
Supplement: Supplementary file 3 [file Table3.docx]

**Supplementary Table S3** The occurrence of proximal phosphorylatable amino acids close to motifs appearing in the differential analysis is shown here. The motifs are sorted in the decreasing order of occurrence in the UCPS-ATG group.

| **Motifs** | **UCPS-ATG (202)** | | **CPS (1576)** | |
| --- | --- | --- | --- | --- |
|  | Number | % | Number | % |
| DEE | 83 | 41.09 | 213 | 13.52 |
| EEE | 79 | 39.11 | 140 | 8.88 |
| KKS | 68 | 33.66 | 112 | 7.11 |
| AKK | 65 | 32.18 | 95 | 6.03 |
| KES | 64 | 31.68 | 155 | 9.84 |
| KAL | 62 | 30.69 | 205 | 13.01 |
| GKK | 60 | 29.70 | 177 | 11.23 |
| DDE | 59 | 29.21 | 169 | 10.72 |
| EKR | 59 | 29.21 | 132 | 8.38 |
| KER | 56 | 27.72 | 96 | 6.09 |
| KKP | 56 | 27.72 | 96 | 6.09 |
| AEK | 55 | 27.23 | 141 | 8.95 |
| EAK | 54 | 26.73 | 130 | 8.25 |
| KAV | 54 | 26.73 | 138 | 8.76 |
| ASK | 51 | 25.25 | 115 | 7.30 |
| KVT | 51 | 25.25 | 174 | 11.04 |
| DED | 47 | 23.27 | 99 | 6.28 |
| GSK | 46 | 22.77 | 219 | 13.90 |
| APK | 46 | 22.77 | 125 | 7.93 |
| EGE | 39 | 19.31 | 116 | 7.36 |
| DKK | 36 | 17.82 | 111 | 7.04 |
