# Supplementary material for: Early Bioinformatic Implication of Triacidic Amino Acid Motifs in Autophagy-Dependent Unconventional Secretion of Mammalian Proteins
Source: Front Cell Dev Biol. 2022 May 13;10:863825. doi: 10.3389/fcell.2022.863825 (PMC9136135; doi:10.3389/fcell.2022.863825)
Supplement: Supplementary file 4 [file Table4.docx]

**Supplementary Table S4.** The structural distance between the triacidic motif and the LIR region in some of the class I proteins from the UCPS-ATG positive dataset, when there is no sequence proximity, is shown.

| **Class I Proteins** | **Uniprot** | **PDB** | **Chain** | **LIR** | **Triacidic Motif** | **Distance between LIR and Triacidic Motif (Å)** |
| --- | --- | --- | --- | --- | --- | --- |
| MAP1LC3B | Q9GZQ8 | 1V49 | A | QAFFLL | DED | 11.5 |
| MTR | Q99707 | 4CCZ | A | RAYHLL | DEE | 3 |
| DSG2* | Q14126 | 5ERD | A, B | ARYVKL | DED | 8.1 |
| CPSF6 | Q16630 | 3Q2S | C, D | KGFALV | DED | 7.1 |
| CDC37 | Q16543 | 5FWL | E | SVWDHI | DDE | 6.6 |
| EEF1D | P29692 | 2N51 | A | AAFNKI | EDD | 7.3 |
| SF3A1 | Q15459 | 6FF7 | EB | VAYAQI | EEE | 7.8 |
| DDX39B | Q13838 | 1XTK | A | VFFGGL | DEE | 4.6 |

*Contains leader peptide
